# Antimicrobial Activity of Selected Banana Cultivars Against Important Human Pathogens, Including Candida Biofilm

**DOI:** 10.3390/foods9040435

**Published:** 2020-04-04

**Authors:** Ramin Saleh Jouneghani, Ana Hortência Fonsêca Castro, Sujogya Kumar Panda, Rony Swennen, Walter Luyten

**Affiliations:** 1 Department of Biology, Katholieke Universiteit Leuven, 3000 Leuven, Belgium; r.saleh.j@gmail.com (R.S.J.); acastro905@gmail.com (A.H.F.C.); walter.luyten@kuleuven.be (W.L.); 2 Plant Physiology and Biochemistry, Universidade Federal de São João Del-Rei, Av. Sebastião Gonçalves Coelho, 400-Chanandour, 35501-296 Divinópolis, MG, Brazil; 3 Mayurbhanj Biological Research (MBR), Bhanjpur, Baripada, 751002 Odisha, India; 4 International Institute of Tropical Agriculture, Arusha 447, Tanzania; rony.swennen@kuleuven.be; 5 Laboratory of Tropical Crop Improvement, Division of Crop Biotechnics, Katholieke Universiteit Leuven, 3001 Leuven, Belgium; 6 Bioversity International, 3001 Leuven, Belgium

**Keywords:** antimicrobial activity, banana cultivars, biofilm inhibitory concentration, food-borne pathogens, *Musa* spp.

## Abstract

Ten banana (*Musa* spp.) cultivars were studied for their antimicrobial properties. Three plant parts (corm, pseudostem and leaves) were collected separately and extracted with different solvents, viz., hexane, acetone, ethanol and water. The 50% inhibitory concentration (IC_50_) was evaluated using a broth microdilution assay. Eight human bacterial and one fungal pathogen were tested. Acetone and ethanol extract(s) often exhibited significant antimicrobial activity, while hexane extracts were less active. Aqueous extracts often showed microbial growth, possibly by endophytes. Leaf extracts were most active, followed by pseudostem, and corm was least active. All the tested banana cultivars were found to contain antimicrobials, as demonstrated by inhibition of selected human pathogens. However, cultivars such as Dole, Saba, Fougamou, Namwah Khom, Pelipita and Mbwazirume showed a broad-spectrum activity, inhibiting all tested pathogens. Other cultivars such as Petit Naine and Kluai Tiparot showed a narrow-spectrum activity, including antibiofilm activity against *Candida albicans*. Our results support the use of different parts of banana plants in traditional human medicine for infections, including diarrhea and dysentery, and some sexually transmitted diseases, as well as for packaging spoilable materials like food.

## 1. Introduction

Edible plants, including bananas, are crucial parts of biodiversity, and their sustainable exploitation has become a valuable sustenance strategy. Recently, consumer awareness is increasingly favoring natural food products, and there is a growing concern about antimicrobial resistance. Secondary metabolites from plants serve as a defense mechanism against pests and pathogens but may also be useable for other (e.g., medical) applications. As antimicrobial resistance is rapidly rising, new approaches are essential to fill the gap in antimicrobial drug discovery. Among several strategies, searching for novel antimicrobial compounds in plant extracts is attractive, and the chemical diversity of natural products is huge. There is also a great potential for plant extracts as food additives because of their antimicrobial, antioxidant and health-related properties [1]. Not only are they generally recognized as safe, but they are typically inexpensive and provide an easy method to extend the shelf life of packaged food [2]. According to the World Health Organization (WHO), unsafe food due to microbial contamination or chemical substances causes more than 200 diseases (https://www.who.int/news-room/fact-sheets/detail/food-safety). One tenth of the world population gets ill every year as a result of eating contaminated food, causing 420,000 deaths annually. The problem is most severe in children under five years old, where diarrheal diseases are the most common. Food spoilage bacteria cause significant food loss with economic, social and environmental impacts [3]. When it comes to treating microbial pathogens, especially in infectious diseases, the scientific evaluation of medicinal plants using ethnobotanical knowledge can offer attractive possibilities for drug discovery [4]. Traditional knowledge includes data such as toxicity, restrictions on use (age, gender), key ingredients(s) for the herbal preparation, which plant parts to be used, mode of preparation (cold/hot, decoctions, maceration), mode of application (external, internal, oral), harvest time, storage conditions, etc. All these parameters help to choose the best starting material for identifying bioactive compounds. A prime example is the study by the Nobel prize winner Professor Tu Youyou on the yield of artemisinin when prepared in the traditional manner (cold water maceration) instead of using hot water extraction [5].

Several parts of the banana such as leaf, pseudostem, flowers, fruit or peel have long been used in traditional medicine in America, Asia and Africa [6]. Recently, several authors reviewed the use of banana plants in traditional medicine across the world [7,8,9]. Traditionally, stem juice, flowers and fruits of the banana plant are used commonly for the treatment of diarrhea and dysentery [10,11]. Oral administration of juice extracted from pseudostem helps in diarrhea among tribal peoples of the Similipal Biosphere Reserve (tribal healer, personal communication). Additionally, the juice is also used in children for treating ulcers and aphthae (https://en.wikipedia.org/wiki/Aphthous_stomatitis) [7]. A mixture of plants, containing equal quantities of roots of *Stereospermum chelonoides*, latex of *Calotropis gigantea* and stem juice of *Musa paradisiaca,* mixed with pepper is given as a decoction for relief from snakebites [12]. The juice extracted from banana leaves is used in the treatment of fresh wounds, cuts and insect bites [13]. Banana leaves make an excellent alternative wound dressing [14,15]. Cold infusions prepared from the roots of *Musa paradisiaca* are used in the treatment of sexually transmitted diseases [16]. In South-East Asian and other cultures, banana leaves are widely used as food wrapping as well as serving plates [17].

To assess ethnomedicinal claims from banana, it is essential to study the antibacterial activity against relevant pathogens. Most previous studies of antimicrobial properties of banana pertain to a single popular cultivar, and often focus on the use of one plant part [16,18,19,20,21,22,23,24]. Therefore, we designed a study of the antimicrobial activity of different parts of selected banana cultivars against food-borne and clinically important pathogens, including *Candida albicans* biofilm.

## 2. Materials and Methods

### 2.1. Sampling

Leaves, pseudostems and corms of nine cultivars of adult banana plants were collected in March, 2015 in the tropical greenhouse from the Laboratory of Tropical Crop Improvement, KU Leuven, Heverlee Campus (Belgium). One cultivar (Petite Naine) was obtained from Africa (Table 1). The greenhouse plants were grown in DCM pot soil type 7.

### 2.2. Extract Preparation

The plant parts (e.g., leaves, pseudostems and corms) were cut into small slices, and dried in an oven at 70 °C until dryness (1–3 days). Samples were then ground to a fine powder using a high-powered HK-10B plant mill (Guangzhou Xulang Machinery & Equipment Co. Ltd., Guangzhou, China). Thereafter, samples were stored in a cold room at 4 °C. For small-scale extractions, we followed the same procedure as previously described [25]. One gram of the fine plant powder was extracted in 15 mL conical Falcon tubes with screwcaps using 10 mL of four different solvents (water, ethanol, acetone and hexane) at ambient temperature with the aid of sonication (4 × 15 min over a 24 h period in a Branson sonicator water bath) and repeated vortexing. After one day, the tubes were centrifuged for 10 min at 3500 *g*, and the supernatant transferred in 1 mL aliquots to 1.5 mL Eppendorf tubes. After evaporation of water and ethanol in a Savant SpeedVac Concentrator (Thermo Scientific) and of acetone and hexane at ambient temperature in a fume hood, the dry weight of each sample was determined (Supplementary Table S1). The dried residue of 1 mL extract was re-dissolved in 200 μL of water (for the aqueous extract) or of dimethyl sulfoxide (DMSO) for the organic extracts; these stocks were stored at 4 °C for the subsequent bioassays.

### 2.3. Antimicrobial Activity

#### 2.3.1. Microorganisms

The bacterial strains used in this study were: *Bacillus cereus* (DPMB 1), *Micrococcus luteus* (DPMB 3), *Staphylococcus aureus* (ATCC 6538, Rosenbach), *Streptococcus faecalis* (DPMB 4)*,* (all Gram-positive), *Aeromonas hydrophila* (ATCC 7966), *Escherichia coli* (Top 10 Invitrogen), *Salmonella enterica* subsp. *enterica* (ATCC 13076) and *Shigella sonnei* (LMG 10473) (all Gram-negative). One fungal strain was used: *Candida albicans* (SC5314). The (frozen) storage, maintenance and preparation of working culture suspensions were carried out in accordance with established procedures described in previous studies [26,27].

#### 2.3.2. Antibacterial Assay (Broth Microdilution Method) and Determination of Inhibitory Concentration (IC_50_)

Antimicrobial activity was assessed as described previously [26] using a broth microdilution method. A two-fold serial dilution series (up to 64-fold) of an extract was prepared in a separate 96-well conical bottom (V) microplate using DMSO. Ten µL of the test sample was transferred into the wells of a test plate, as well as the positive control (ciprofloxacin, stock 200 µg/mL) and blank (solvent) controls (5% final concentration DMSO or water). Wells of a microdilution plate were inoculated with 190 µL of a diluted standardized inoculum in Mueller-Hinton/ Luria Bertani broth for bacteria such as *A. hydrophila, S. enterica* and *S. faecalis* (Optical Density OD = 0.003 at 620 nm). Control wells were prepared with 190 µL sterile Mueller-Hinton/ Luria Bertani broth plus 10 µL extract, to correct for any absorption due to extract components. The microdilution plates were placed in a shaker-incubator at 37 °C for 18 h, and then read on a Mithras LB 940 Multimode Microplate Reader (Berthold Technologies GmbH & Co. KG, Bad Wildbad, Germany) at 620 nm with lamp energy of 13,000 using the MikroWin 2000 software package. The OD was measured at a wavelength of 620 nm, and wells with a plant extract were corrected for the absorption contributed by the extract. Tests were typically carried out in duplicate. The relative inhibition (%) of the test sample was calculated as 100% − {[(OD_test sample_-OD_extract control_) × 100%]/OD_solvent control_}. Data from dose-response experiments were represented as the percentage of inhibition and analyzed with Prism™ (GraphPad Prism 5.0 Software Inc., San Diego, CA, USA). The IC_50_ for each growth condition was calculated by fitting the data to a non-linear least-squares sigmoid regression curve, keeping the minimum and maximum fixed at 0 and 100%, respectively. The positive control ciprofloxacin showed 100% inhibition against all test organisms, while the negative controls (5% DMSO) did not show any antibacterial activity.

#### 2.3.3. Anti-Candida Activity and Determination of Biofilm Inhibitory Concentration (BIC_50_)

The anti-biofilm activity was determined by following exactly the method described recently [28] on *C. albicans* SC 5314. A two-fold serial dilution (up to 32-fold) of an extract was prepared in a 96-well conical bottom (V) polystyrene microtiter plate. Data from dose-response experiments were represented as the percentage of inhibition compared to control and analyzed in the same way as for the IC_50_. The biofilm IC_50_ (BIC_50_) corresponds to the concentration that would yield an inhibition of 50%. The positive control miconazole showed 100% inhibition, while the negative controls (2% DMSO) did not show any anti-Candida biofilm activity.

### 2.4. Determination of Total Phenolic Content

The total phenolic content (TPC) was estimated using the Folin-Ciocalteu’s reagent according to Ainsworth and Gillespic [29] with slight modifications. Briefly, 10 µL of extract (20 mg/mL) solution was mixed with 10 µL of Folin & Ciocalteu’s phenol reagent 2 M (Sigma) in a 96-well flat-bottom plate. After 5 minutes, 20 µL of Na_2_CO_3_ (10%) was added to the mixture, followed by addition of 60 µL sterile water (MilliQ). After proper mixing, the plates were incubated for 90 minutes at 37 °C. Afterwards, the absorbance was measured at 765 nm (Infinite M200 spectrophotometer) using a mixture of water and reagents as a blank. All experiments were performed in duplicate. A gallic acid dilution series was used to generate a calibration curve (Supplementary Figure S1). The outcome data were expressed as µg/mg of gallic acid equivalents (GAE) in micrograms per milligram (µg GAE/mg) of extract.

### 2.5. Genetic Relationship Analysis Using Distance-Based Methods

Nine out of ten cultivars originated from the largest international *ex situ* collection of banana germplasm (International Transit Centre, ITC) maintained by Bioversity International, and hosted at the Catholic University in Leuven (KU Leuven), Belgium. Christelova and co-workers [30] summarized the results of systematic cytological and molecular characterization of this entire *Musa* collection. For our analysis, we used their ITS sequence data to construct a phylogenetic tree with a Neighbor Joining (NJ) method using SplitsTree4 v4.1.11 (Universität Tübingen, Tübingen, Germany https://uni-tuebingen.de/fakultaeten/mathematisch-naturwissenschaftliche-fakultaet/fachbereiche/informatik/lehrstuehle/algorithms-in-bioinformatics/software/splitstree/) based on the Jukes-Cantor and uncorrected *p*-distances [31]. 

### 2.6. Statistical Analysis 

All IC_50_ values were obtained with Prism™ (GraphPad Prism 5.0 Software Inc., San Diego, CA, USA) by fitting the data to a non-linear least-squares sigmoid regression curve. Spearman *r* were analyzed using GraphPad Prism, and statistical significance was assumed with 95% confidence interval, *p*-value (two tailed) = 0.05. Principal component analysis and heat maps were constructed using ClustVis, a web tool for visualizing clustering of multivariate data (BETA) (https://biit.cs.ut.ee/clustvis/). Unit variance scaling was applied to the rows of this table; SVD (Singular Value Decomposition) with imputation was used to calculate principal components.

## 3. Results

### 3.1. Effect of Solvent

**Figure 1:**
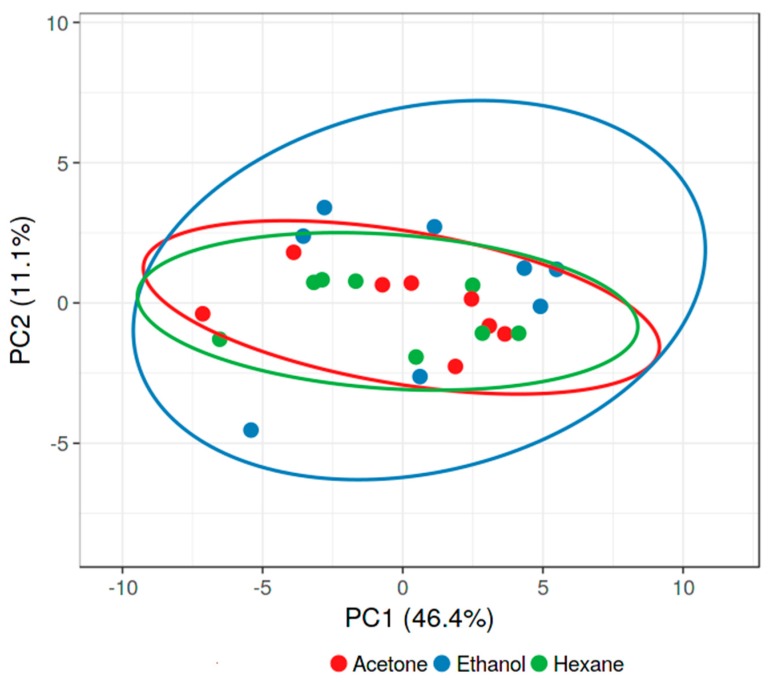
Principal component analysis of antibacterial activity (prediction ellipses with probability 0.95, *N* = 24 data points).

**Figure 2:**
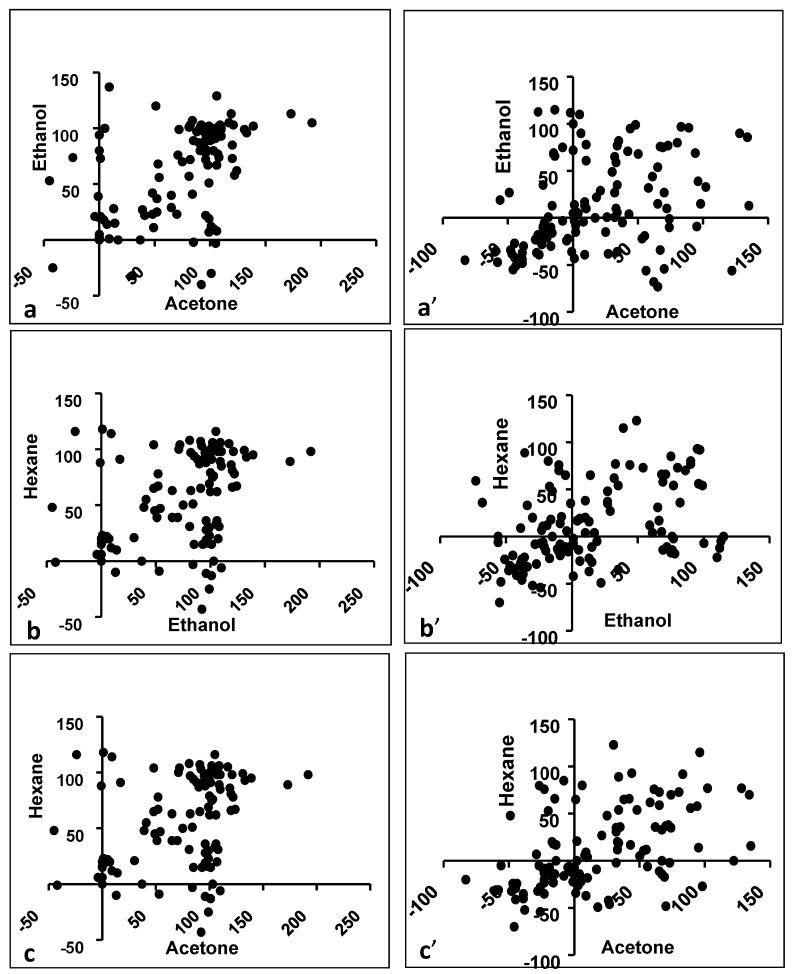
Antimicrobial activity correlation between extracts in different solvents for Gram-positive and Gram-negative bacteria. (**a**) Acetone vs. Ethanol (Gram-positive), (**a**’) Acetone vs. Ethanol (Gram-negative), (**b**) Ethanol vs. Hexane (Gram-positive), (**b**’) Ethanol vs. Hexane (Gram-negative), (**c**) Hexane vs. Acetone (Gram-positive), (**c**’) Hexane vs. Acetone (Gram-negative).

Antibacterial activity against eight bacterial pathogens was studied for extracts prepared in four different solvents: nonpolar (hexane), aprotic polar (acetone), protic polar (ethanol) and polar (water). The antibacterial inhibitory activity (%) varied with the solvent used to prepare an extract, although all three organic solvents often exhibited significant antibacterial activity (Figure 1; Table 2; Supplementary Figure S2A–D). The X and Y axis show principal component 1 and 2, that explain 46.4% and 11.1% of the total variance, respectively (Figure 1). Both acetone and hexane appear equally effective solvents in that the same extracts are active, but active ethanol extracts are regularly found in other samples. This implies that ethanol does not appear to extract the same bioactive compound(s) as acetone/hexane. Indeed, there is higher correlation between the bioactivity of the hexane versus acetone (Spearman *r* = 0.5198) than the ethanol versus hexane (Spearman *r* = 0.3523) extracts (Table 3; Figure 2a–c). Surprisingly, however, the acetone versus ethanol correlation is high (Spearman *r* = 0.5105).

Aqueous extracts often showed much growth in the control wells, possibly due to endophytes, thus rendering interpretation difficult. Therefore, the aqueous extracts were boiled, filtered (0.22 µm) and retested against *S. aureus*. Only in a few cases could antimicrobial activity be demonstrated after this treatment, e.g., Fougamou-pseudostem-aqueous extract exhibited 100% activity against *S. aureus*.

### 3.2. Total Phenolic Contents of Different Extracts

**Figure 3:**
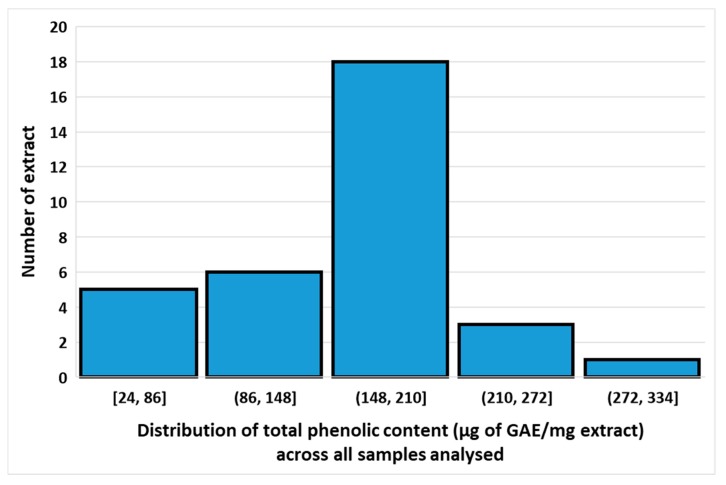
Distribution of total phenolic content across all samples analysed.

The total phenolic content (TPC) of samples was calculated from the regression equation of the gallic acid calibration curve (*R*^2^ = 0.9902), expressed in gallic acid equivalents (GAE) as µg/mg of the crude extract (Supplementary Figure S1). There is over a ten-fold variation in TPC between extracts in various solvents of different cultivars (24 to 309 µg of GAE/mg extracts) (Supplementary Table S2), although most extracts show values between 148 and 210 µg of GAE/mg extract (Figure 3). Since significant amounts of TPC were found in three quite different solvents, it can be concluded that the cultivars probably contain a mixture of phenolic compounds, ranging from very hydrophobic ones to more polar ones. Across all samples, TPC correlates best between acetone and hexane extracts (Spearman *r* = 0.91), better than between acetone and ethanol extracts (*r* = 0.57), whereas the lowest correlation is between ethanol and hexane extracts (*r* = 0.43). There is a good correlation between the corresponding extracts of leaf versus pseudostem (*r* = 0.82), suggesting that the TPC in most cultivars does not differ very much between plant parts. Among the different cultivars with a fairly high TPC (>200 µg of GAE/mg extract), several extracts showed broad-spectrum antibacterial activity: Fougamou-pseudostem-ethanol (TPC = 301.1 µg/mg, active against *B. cereus, M. luteus, S. aureus, S. faecalis* and *E.coli*), Cachaco-leaf-acetone (TPC = 246.3 µg/mg, active against *B. cereus, M. luteus, S. aureus, S. faecalis* and *S. enterica*), Giant Cavendish-leaf-ethanol (TPC = 236.8 µg/mg, active against *B. cereus, M. luteus, S. faecalis* and *S. enterica*) and Giant Cavendish-leaf-acetone (TPC = 213.32 µg/mg, active against *B. cereus, M. luteus, S. aureus, S. faecalis* and *S. enterica*) (Supplementary Table S2).

### 3.3. Influence of Plant Part for Gram-Positive Activity

**Figure 4:**
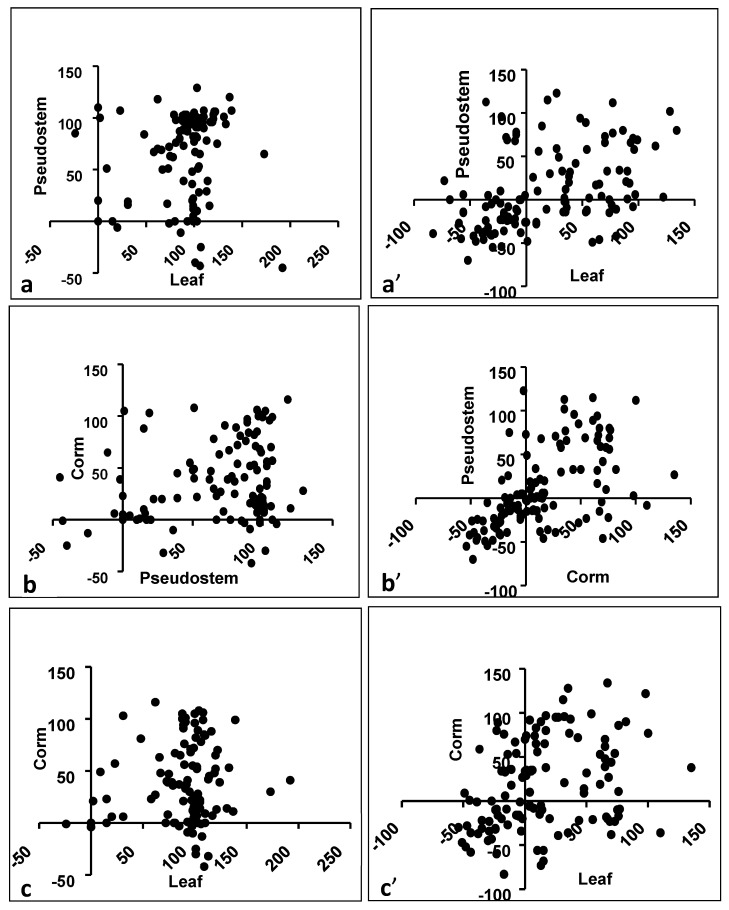
Correlation between different plant parts. (**a**) Leaf vs. Pseudostem (Gram-positive), (**a**’) Leaf vs. Pseudostem (Gram-negative), (**b**) Pseudostem vs. Corm (Gram-positive), (**b**’) Pseudostem vs. Corm (Gram-negative), (**c**) Leaf vs. Corm (Gram-positive), (**c**’) Leaf vs. Corm (Gram-negative)

The antibacterial activity among Gram-positives depended on the plant part; leaf and pseudostem extracts were most often active, while corm extracts had activity in fewer cases. It could be assumed that if a bioactive compound is produced in one plant part, then the chance is high that it would also be produced in the immediately connecting part of the same plant. Indeed, the bioactivity in a leaf is fairly correlated with that of the pseudostem (Spearman *r* = 0.2345), and that of the pseudostem with the corm (Spearman *r* = 0.2439) (Table 3; Figure 4a–c). The bioactivity in the corm is not highly correlated with that of a leaf (Spearman *r* = 0.1172).

### 3.4. Effect of Solvent for Activity Against Gram-Positives

Acetone and ethanol extracts revealed similar activity scores against *B. cereus* and *S. faecalis* for leaf and pseudostem but showed different activity patterns against *M. luteus* and *S. aureus* (Table 2; Supplementary Material Figures S1 and S2A–I). *Bacillus cereus* was found to be inhibited to the greatest extent by extracts in the different organic solvents tested. 

### 3.5. Effect of Plant Parts for Activity Against Gram-Negatives

All the banana cultivars were also tested against four Gram-negative bacteria, and several extracts showed pronounced antibacterial activity (>50% of growth inhibition) for different plant parts (Table 4; Supplementary Figure S2F–I). The antibacterial activity against Gram-negative bacteria depended on the plant part; leaf extract was most often active, while pseudostem and corm extracts were less often active. But when compared by plant part, the bioactivity in a leaf is significantly correlated with that of the pseudostem (Spearman *r* = 0.4936), and that of the pseudostem with the corm (Spearman *r* = 0.6321), whereas the correlation of a leaf with the corm is lower (Spearman *r* = 0.3396) (Table 3; Figure 4a’–c’). This is similar to the pattern for Gram-positives. 

### 3.6. Effect of Solvent for Activity Against Gram-Negatives

Surprisingly, the bioactivity correlated between all three organic solvents, i.e., acetone versus ethanol (Spearman *r* = 0.4070), ethanol versus hexane (Spearman *r* = 0.4316) and hexane versus acetone (Spearman *r* = 0.5326) extracts (Table 3; Figure 2a’–c’).

### 3.7. Effect of Bacterial Strain

The activity spectrum depended on the test bacterium. In general, Gram-positives were more susceptible than Gram-negatives. Amongst Gram-positives, *S. aureus* was found to be more resistant, while the other three bacteria were sensitive to most extracts. However, amongst Gram-negatives, susceptibility varied with strains. *A. hydrophila* was the least susceptible strain, followed by *E. coli*. Most extracts were active against *S. enterica* and *S. sonnei,* but at higher concentration compared with Gram-positives.

With two-fold serial dilution, only few extracts showed activity against Gram-negatives, such as Fougamou-leaf-acetone (99 µg/mL), Dole-leaf-acetone (107 µg/mL), and Mbwazirume-leaf-acetone (152 µg/mL) against *S. sonnei*. A few other extracts also showed activity, but with higher IC_50_ (1000 µg/mL) against *A. hydrophila* such as Kluai Tiparot-pseudostem-ethanol (1354), Dole-leaf- acetone (1973 µg/mL) and Cachaco-leaf-ethanol against *E. coli* (1787 µg/mL) (Table 5).

### 3.8. Study of Antibacterial IC_50_ Values

The IC_50_ value of selected organic solvent extracts was also determined against all test pathogens using a two-fold serial dilution (Table 5, Supplementary Figure S3A–D). The IC_50_ value of aqueous extracts were not calculated because most of the extracts’ correction OD did not decrease on further serial dilution, and when streaked out on solid media showed microbial growth. This may be due to the presence of endophytes, or contamination during preparation of the aqueous extracts. From the serial dilution results, it is clear that the most potent extracts (IC_50_ < 100 µg/mL) are acetone extracts from the leaves of Mbwazirume (53 µg/mL), Dole (61 µg/mL) and Saba (99 µg/mL). Notably, two ethanol extracts of leaves (Mbwazirume and Cachaco) also showed a strong inhibitory activity against *B. cereus* (130 µg/mL and 159 µg/mL, respectively). When comparing activity against *B. cereus* with that against *S. aureus*, the latter shows a lower sensitivity (all IC_50_s >200 µg/mL). Nevertheless, fairly potent against *S. aureus* is the acetone extract of the leaf of Saba (373 µg/mL) and Pelipita (442 µg/mL). A few additional cultivars were able to inhibit growth of *M. luteus,* including Namwah Khom-leaf-acetone (31 µg/mL), Mbwazirume-leaf-acetone (33 µg/mL) and Dole-pseudostem-acetone (121 µg/mL). Interestingly, leaf parts of most cultivars extracted with acetone are active against *S. faecalis* with low IC_50_s (<100 µg/mL): (Kluai tiparot (28), Namwa Khom (31), Saba (37), Pelipita (58), Mbwazirume (83) and Dole (53) (Table 5). 

### 3.9. Antifungal Activity Against Candida Biofilm

All crude extracts of different banana cultivars were also evaluated in an anti-biofilm assay against *C. albicans*. Ethanol-, acetone- and to a lesser extent hexane extracts showed anti-biofilm activity (Table 6, Supplementary Figure S3E). None of the aqueous extracts showed anti-biofilm activity against *C. albicans*.

**Figure 5:**
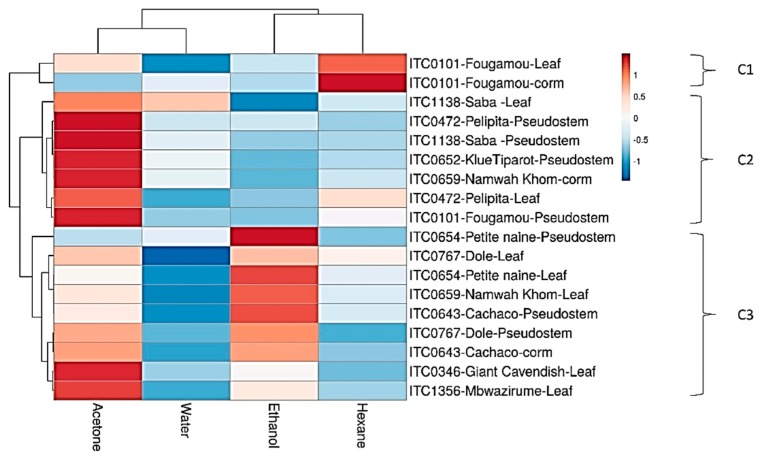
Heat map and clustering of *Candida* anti-biofilm activity of extracts from banana cultivars. C1–C3, Different clusters.

A heat map of these results is shown in Figure 5. Interestingly, Fougamou-leaf and corm represent an isolated cluster (C1) due to its high activity in the hexane extract combined with variable activity in the acetone extract, while no activity was seen in the water and ethanol extracts.

The neighboring cluster (C2) has strong activity in the acetone extract, but little in the other extracts. It contains mostly extracts of pseudostem, i.e., Saba, Pelipita, Klue Tiparot and Fougamou, as well as leaf extracts (Saba, Pelipita) and Namwa Khom corm. The last cluster (C3) shows (from top to bottom) decreasing activity in the ethanol extracts, while that in the acetone extracts generally increases (there is no activity in the water and hexane extracts). This cluster may be separated into two subgroups: Petite Naine-pseudostem and leaf, Dole-leaf, Namwa Khom-leaf and Cachaco-pseudostem (at the top); and Dole-pseudostem,-Cachaco-corm, Giant Cavendish-leaf and Mbwazirume-leaf (at the bottom). These patterns strongly suggest that each cluster contains a different antifungal compound. Furthermore, a serial dilution was performed for a few extracts that showed >60% inhibition of biofilms, and the BIC_50_ values are presented in Table 6. The most potent extracts are the ethanol extract from leaves of Namwah Khom (31 µg/mL), Dole (51 µg/mL) and Fougamou (220 µg/mL). Equally, extracts from pseudostem, such as the ethanol extract of Petite Naine (44 µg/mL) and the acetone extracts of Saba (177 µg/mL) and Klue Tiparot (183 µg/mL) strongly inhibit the formation of *Candida* biofilms.

## 4. Discussion

### 4.1. Microbial Strains Used

Numerous studies have reported antimicrobial activity in *Musa*. The most common bacterial strains used for these tests were *E. coli*, *Pseudomonas aeruginosa, Salmonella typhi, Shigella dysenteriae, B. cereus* and *S. aureus*, although many reports use only a small number of these [19,20,21,22,23,33,34]. The present study includes most of these strains, in addition to several others. We also include some food-borne pathogens, so that our results are relevant for the treatment of infectious diseases, as well as for the prevention of contamination in food (e.g., by using banana leaves for packaging and storing food). In addition, we tested *C. albicans* biofilms. Although extracts from all our cultivars show activity against Gram-positives, for Gram-negative pathogens the antibacterial profile depends more on the extraction solvent as well as the plant parts. This may be due to a lack of permeability of the lipopolysaccharide outer membrane of Gram-negatives, and many previous studies have already reported similar observations [27,35,36].

More interestingly, a few of our extracts inhibit *C. albicans* biofilm. Most emerging pathogens have the ability to form biofilms to ensure successful colonization and survival in host tissues. Pathogens in these biofilms tend to be much less sensitive to antimicrobials, and this poses serious problems for treatment. 

### 4.2. Effect of Solvent on Antibacterial Activity

The antimicrobial activity observed depends on the extraction solvent, presumably due to differential solubility of the bioactive compounds with solvent polarity [36,37]. Both acetone and ethanol extracts of banana leaves and pseudostem show the strongest antibacterial activity, followed by hexane extracts. The differences in the activity profiles are probably due to the capability of the extracting solvent to dissolve the bioactive compounds depending on their polarities. According to Padam and co-workers [38], methanol served as a superior solvent in the extraction of antimicrobial and antioxidant compounds from banana (*Musa paradisiaca* cv. Mysore) inflorescence. We find similar results for other plant parts where ethanol extracts have similar antimicrobial activity as acetone extracts, and even better activity when compared to hexane. Nevertheless, the presence of bioactive compounds also depends on handling and preparation of the plant materials during harvesting and drying [39], on the extraction procedure (sample-to-solvent ratio, extraction time and temperature), environmental conditions [40] and geographical origin [41].

### 4.3. Aqueous Extract Problems

Aqueous extracts often showed significant microbial growth in the control wells, rendering interpretation difficult. The contamination of aqueous extracts by *Musa* endophytes has been documented [32]. When our aqueous extracts were boiled, filtered (0.22 µm) and retested against *S. aureus* antimicrobial activity could only be measured in only a few cases (e.g., Fougamou pseudostem aqueous extract exhibited 100% activity against *S. aureus*). Surprisingly, in most cases aqueous extracts caused no problems in the *C. albicans* biofilm test. This may be because banana endophytes do not grow well in the medium used for Candida biofilm tests, or are inhibited by *C. albicans*, or are removed during washing steps of the biofilm formation assay.

Overall, our results indicate that water extraction was less likely to dissolve antimicrobial compounds, so that a highly polar solvent may seem a poor choice. Nonetheless, it is the solvent mostly used by indigenous tribal peoples. They have of course far less access to a range of solvents (although some are known to use fermented beverages containing ethanol). Moreover, they often use boiling water, whereas our aqueous extracts are prepared at ambient temperature. Since solubility in general increases with temperature, boiling may extract more compounds in water compared to our procedure (which uses ambient temperature, albeit combined with sonication).

In some cases, where banana parts (e.g., leaves) are closely apposed to a target site (e.g., a wound), it is conceivable that more hydrophobic compounds could diffuse from the banana part into the patient and exert (at least a local) effect. Likewise, when used as packaging, lipophilic compounds may diffuse from the banana leaf into the food, especially if this is greasy.

### 4.4. Effect of Plant Part on Antibacterial Activity

Literature reviews indicate that banana fruits and flowers contain antibacterial principles, but no reports are available for antibacterial activities from the corm of banana plants [42]. We detected antibacterial activity in corm, but it is clear that leaf and pseudostem contain stronger antibacterial activity compared to corm. Leaves and pseudostem are above ground plant parts and can be easily harvested, unlike the corm. In addition, a banana plant produces continuously new leaves until fruit harvest, so that repeated harvesting of leaves is feasible. A recent study by Siddique et al. [43] finds antimicrobial activity from peels of *Musa sapientum*. Although the concentration of metabolites may differ considerably between plant parts, it is reasonable to assume that the presence of a bioactive compound in one plant part of a variety renders its presence much more likely in neighboring parts of the same variety. This is also what we observe: the activity correlation is better for plant parts that are closer (leaf and pseudostem, pseudostem and corm) than for more distant ones (leaf and corm).

### 4.5. Antimicrobial Activity Patterns and Their Implications for Bioactive Compounds

In several cases, the antimicrobial activity patterns seen with extracts from different plant parts, or different cultivars, or prepared in different solvents, suggested the presence of multiple bioactive compounds. Until they are isolated, this is of course speculative, but it is consistent with the findings in many other medicinal plants, where the presence of multiple compounds with similar bioactivity is the rule rather than the exception. Hexane extracts from leaves of all our cultivars show a similar activity profile against *B. cereus* and *S. faecalis*, suggesting that the chemical composition of the lipophilic compounds of different banana cultivars may be similar, at least for those compounds responsible for this antibacterial property. Villaverde et al. [44] carried out a GC–MS analysis of the lipophilic extracts of the banana peels from different cultivars and concluded that the main components are sterols, followed by fatty acids, aliphatic alcohols and α-tocopherol. These phytoconstituents are thought to be mostly responsible for the antimicrobial properties [45]. A few minor constituents such as steryl glucosides, campesteryl 3-β-D-glucopyranose, stigmasteryl 3-β-D-glucopyranoside and sitosteryl 3-β-D-glucopyranoside are known as antimicrobial and antiprotozoal compounds in several plants [46,47], but it is not clear whether they are present at sufficient levels in banana parts to contribute to their antimicrobial activity. The same goes for polyphenols (see Section 4.7).

In most cases, banana extracts contain complex mixtures of compounds from many chemical groups. The antimicrobial activity we measured may well reflect the combined effects of several bioactive molecules. Although some of our extracts already showed impressive potency, their activity may even increase when purified fractions or compounds are tested. In the present work, we have covered only the tip of the iceberg in our understanding of the antimicrobial activity of these banana cultivars. Work is in progress on the isolation and identification of the bioactive compounds using bioassay-guided purification.

### 4.6. Effect of Cultivar on Antibacterial Activity

There are several reports on screening for antibacterial properties of specific local banana cultivars [16,18,19,20,21,22,23,24]. However, few compare a broad range of cultivars as we did. We can therefore ask the question whether the antimicrobial activity profile correlates with genetic relatedness.

**Figure 6:**
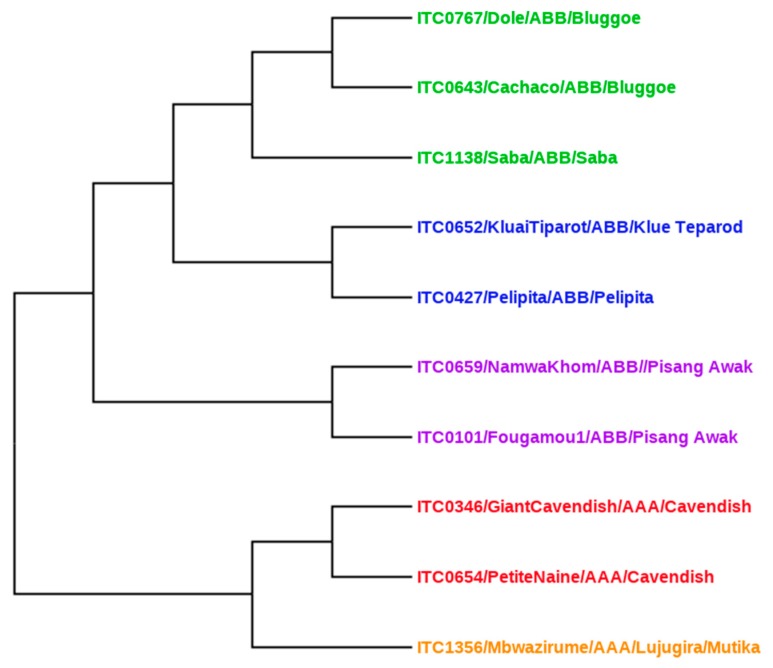
Construction of Neighbor Joining (NJ) tree using all test cultivars.

Assuming that the metabolomes of two plant cultivars will resemble each other more the closer the cultivars are related genetically, we expect a correlation between the bioactivity of a banana variety and its degree of relatedness. For ease and clarity of the discussion, a phylogenetic tree with the Neighbor Joining (NJ) method was constructed (Figure 6). Using all the test accessions, the tree distributed into two main clades ABB (top) and AAA (bottom) (Figure 6). The ABB clade is nicely correlated with antibacterial activity, and the cultivars with genome ABB were also found to be effective for anti-biofilm activity (see heat map Figure 5). The top cluster contains almost all ABB genome cultivars, i.e., Fougamou, Saba, Pelipita, Klue Tiparot, Namwa Khom and Fougamou. The bottom cluster comprises mostly cultivars with an AAA genome, i.e., Giant Cavendish, Petite Naine and Mbwazirume, in addition to some ABB cultivars (Cachaco, Dole and Namwa Khom).

### 4.7. Role of Phenolic Compounds in Observed Biological Effects 

In recent years, polyphenols have received a great deal of attention due to their bioactivity. They are found in a wide variety of fruits as well as vegetables, and their consumption is beneficial for human health [48]. There are different classes of polyphenols known as flavonoids, phenolic acids, lignans and stilbenes; many have been reported as antimicrobials. A range of solvents has been used to extract polyphenols from plant material, such as water, methanol, methanol/formic acid, methanol/water/acetic or formic acid, etc. [49]. Solvents with different polarity can extract individual polyphenols to different degrees, and this could account for different antimicrobial activities of the extracts. That argument would of course also apply to non-polyphenolic antimicrobial compounds. Ethanol is one of the solvents that we used, and it is said to be the best for extracting polyphenols [50].

Often, polyphenols are quantified by their gallic acid equivalent (GAE). We estimated TPC in a number of our crude extracts that showed strong antimicrobial activity (Supplementary Table S2). There is more than 20-fold difference in TPC among extracts, and even extracts with the lowest TPC showed activity against some bacteria. However, phenolic compounds may contribute significantly to antibacterial bioactivity, as most extracts with high TPC also showed broad-spectrum antimicrobial activity. Nonetheless, further studies are needed using bioassay-guided purification to identify the active compound. Vasco found that “the content of polyphenols in *Musa cavendish* peel was similar to *Rubus glaucus* Benth (7300 mg/100 g sample, fresh basis (f.b.)) and *V. floribundum* Kunth (3000 mg/100 g sample, f.b.) which are considered to have high levels of phenolic compounds [51]”. Borrero and Santacruz [52] found no statistically significant difference in the GAE content between three *Musa* varieties. Phenolic profiles in the pulp and peel of nine plantain cultivars (*Musa* spp.) were also investigated by LC/HRMS: “the pulp phenolic profile was dominated by hydroxycinnamic acids, whereas the peel phenolic profile was dominated by flavonols” [53]. However, quantification of phenolic compounds revealed no big difference among nine plantain cultivars [53]. Similar results were also observed by Aquino et al. with variation among the different plant parts, but not so much among the cultivars, viz., TPC among 15 cultivars ranged from 23.15 to 33.28 mg/100 gm GAE for unripe pulp, 42.4 to 77.07 mg/100 gm GAE for ripe pulp, 32 to 61 mg/100 gm GAE for unripe peel and 60.39 to 115.7 mg/100 gm GAE for ripe peel [54]. We find typical values of 160 µg/mg GAE in our crude extracts. Since the yields of the crude extracts are typically 25–75 mg/g dried plant material for the organic solvents, and the weight loss upon drying is typically 90%, this means 40–120 mg/100 g original plant material. These TPC values are in the same range as those reported in the literature.

It is therefore possible that part of the antimicrobial activity that we observed was due to polyphenols, and that variation in polyphenol composition and GAE accounted for differences in antimicrobial activity between different plant parts. However, it seems unlikely that the relatively small TPC differences observed between cultivars would account for their strong differences in antimicrobial activity. Also, the fact that the antimicrobial spectrum may differ considerably between cultivars pleads against a single polyphenol accounting for most of the antimicrobial activity that we observed, but combinations of polyphenols differing in their antimicrobial spectrum could explain our observations.

### 4.8. Application of Banana in the Food Industry and Beyond

Banana is one of the oldest crops cultivated in the history of human agriculture. Padam et al. [55] discuss extensively the breakthrough in the utilization of banana by-products. The authors conclude that “recycling and the utilization of agricultural by-products and waste for the creation of commercially viable and income-generating products is not a new topic” [55]. The genus *Musa* comprises numerous varieties and cultivars, some well-known, but others much less, and there are unlimited possibilities to utilize its different parts: “by-products such as pseudostem, rhizome, leaves, fruit stalks, and peels from the common varieties to some extent are potential raw materials in areas of food and non-food industries, providing each a different application” [55].

There are numerous food-borne pathogens that cause illness and occasionally even death. Elimination of microbes from food without compromising the desirable properties of the product is still a challenge for the food industry [27]. The findings of the current study could pave the way for suitable and natural preservatives for the food-processing industry to control food-borne pathogens. However, more studies are necessary to test these extracts with food matrixes, and to isolate the bioactive compounds using bioassay-guided purification.

### 4.9. Strengths and Limitations of Our Study

There are several reports on the antibacterial activities of some banana plant parts (mostly fruit and peel) in some banana cultivars. The present study, however, deals with a broad selection of banana cultivars and involves the systematic testing of different non-fruit plant parts which are always readily available. Additionally, the use of a range of solvents, activity tests performed with a broth dilution method and the inclusion of food-borne pathogens make our study unique. Nonetheless, more bacteria and fungi could be tested, depending on the intended application in medicine or food. Also, the range of cultivars could be extended. A major limitation is that the antimicrobial compounds remain to be identified.

### 4.10. Conclusions

In conclusion, the present study demonstrates that the genetically closely related banana cultivars with genome ABB have better antimicrobial properties against food-borne and clinically important pathogens. Moreover, the antibacterial activity is stronger in leaves and pseudostem than in the corm. This supports the traditional use of banana extracts for treating infections, including diarrhea and dysentery. Additionally, selected cultivars showed anti-Candida biofilm activity, which is interesting in light of traditional use for treating sexually transmitted diseases. 

## Figures and Tables

**Table 1:** List of banana cultivars studied for antimicrobial activity.

ITC *	Cultivar	Genome	Subgroup
ITC0767	Dole	ABB	Bluggoe
ITC0643	Cachaco	ABB	Bluggoe
ITC1138	Saba	ABB	Saba
ITC0652	Kluai Tiparot	ABB	unknown
ITC0472	Pelipita	ABB	unknown
ITC0659	Namwah Khom	ABB	Pisang Awak
ITC0101	Fougamou	ABB	Pisang Awak
ITC0654	Petite Naine	AAA	Cavendish
ITC0346	Giant Cavendish	AAA	Cavendish
ITC1356	Mbwazirume	AAA	Mutika/Lujugira

* International Transit Centre code.

**Table 2:** Antibacterial activity of selected extracts of banana cultivars against Gram-positive bacteria (relative inhibition in %).

ITC/Cultivar Name/Plant Part	*B. cereus*			*M. luteus*			*S. aureus*			*S. faecalis*		
	A	E	H	A	E	H	A	E	H	A	E	H
ITC0346-Giant Cavendish-leaf	101	99	99	105	112	98	80	0	0	96	133	93
ITC1356-Mbwazirume-leaf	99	131	99	113	113	89	101	109	106	100	102	106
ITC0659-Namwah Khom-leaf	103	121	98	89	100	62	98	93	15	90	102	75
ITC1138-Saba-leaf	97	100	79	85	120	81	101	81	31	90	96	88
ITC0472-Pelipita-leaf	102	139	95	73	120	66	103	92	103	99	101	91
ITC0652-Kluai Tiparot-leaf	99	110	98	62	124	67	100	110	98	93	110	85
ITC0643-Cachaco-leaf	105	117	105	74	−24	116	99	72	104	74	−24	116
ITC0767-Dole-leaf	97	88	91	58	122	78	101	91	107	58	122	78
ITC1356-Mbwazirume-pseudostem	99	101	15	29	65	39	−40	92	−43	80	91	65
ITC0101-Fougamou-pseudostem	101	100	93	78	96	−11	100	110	−6	81	96	89
ITC0659-Namwah Khom-pseudostem	129	106	36	73	1	118	94	0	0	103	82	63
ITC1138-Saba-pseudostem	99	105	103	80	103	76	67	98	16	88	95	97
ITC0472-Pelipita-pseudostem	95	107	98	51	99	69	10	103	0	82	94	89
ITC0652-Kluai Tiparot-pseudostem	12	101	99	70	75	50	77	107	20	87	90	87
ITC0643-Cachaco-pseudostem	95	96	28	−2	85	15	0	17	91	−2	85	15
ITC0767-Dole-pseudostem	98	102	103	67	106	62	102	99	−25	67	106	62
ITC0767-Dole-corm	−3	105	33	23	70	39	−30	101	−13	23	70	39

E—Ethanol, A—Acetone, H—Hexane. Growth inhibition (%) data were rounded to the nearest integer. Single-underlined values represent inhibition above 50%. All four Gram-positive strains exhibited 100% inhibition when tested with ciprofloxacin (20 µg/mL).

**Table 3:** Calculation of nonparametric correlation (Spearman *r*) between different plant parts and solvents.

Parts/Solvents	Parameters	Gram-Positive	Gram-Negative
Leaf vs. Pseudostem	Spearman *r*	0.2345	0.4936
	95% confidence interval	0.0523 to 0.4016	0.3401 to 0.6215
	*p* value (two-tailed)	0.0099	<0.0001
	Is the correlation significant? (alpha = 0.05)	Yes	Yes
Pseudostem vs. Corm	Spearman *r*	0.2439	0.6321
	95% confidence interval	0.0623 to 0.4100	0.5068 to 0.7313
	*p* value (two-tailed)	0.0073	<0.0001
	Is the correlation significant? (alpha = 0.05)	Yes	Yes
Leaf vs. Corm	Spearman *r*	0.1172	0.3396
	95% confidence interval	−0.0687 to 0.2953	0.1655 to 0.4931
	*p* value (two-tailed)	0.2022	0.0001
	Is the correlation significant? (alpha = 0.05)	No	Yes
Acetone vs. Ethanol	Spearman *r*	0,5105	0.4070
	95% confidence interval	0.3599 to 0.6351	0.2406 to 0.5502
	*p* value (two-tailed)	<0.0001	<0.0001
	Is the correlation significant? (alpha = 0.05)	Yes	Yes
Ethanol vs. Hexane	Spearman *r*	0.3523	0.4316
	95% confidence interval	0.1795 to 0.5040	0.2685 to 0.5706
	*p* value (two-tailed)	<0.0001	<0.0001
	Is the correlation significant? (alpha = 0.05)	Yes	Yes
Acetone vs. Hexane	Spearman *r*	0.5198	0.5326
	95% confidence interval	0.3709 to 0.6427	0.3861 to 0.6529
	*p* value (two-tailed)	<0.0001	<0.0001
	Is the correlation significant? (alpha = 0.05)	Yes	Yes

**Table 4:** Antibacterial activity of selected extracts from banana cultivars against Gram-negatives (relative inhibition in %).

ITC/Cultivar Name/Plant Part	*A. hydrophila*			*E. coli*			*S. enterica*			*S. sonnei*		
	A	E	H	A	E	H	A	E	H	A	E	H
ITC0346-Giant Cavendish-leaf	−21	−20	−15	34	77	54	134	86	70	34	35	54
ITC1356-Mbwazirume-leaf	−17	−10	−16	−83	−45	−20	48	99	54	67	−34	33
ITC0654-Petite naine-leaf	95	−9	14	−38	−30	−52	38	−5	65	74	−1	35
ITC0101-Fougamou-leaf	−23	−9	−8	−56	19	−5	128	90	77	56	−56	−6
ITC1138-Saba-leaf	−23	−9	−20	−28	−37	−32	72	10	38	122	−56	0
ITC0472-Pelipita-leaf	−23	−10	76	−1	−36	−22	32	27	35	6	90	82
ITC0652-KlueTiparot-leaf	−20	−17	53	1	−43	−34	83	97	92	65	−73	59
ITC0643-Cachaco-leaf	−16	−16	18	9	−39	9	96	39	115	34	−36	89
ITC0767-Dole-leaf	−27	−18	81	−58	−47	−31	44	95	93	62	−68	36
ITC0346-Giant Cavendish-pseudostem	−23	−17	−23	−2	112	−12	81	81	73	1	−3	−13
ITC1356-Mbwazirume-pseudostem	−24	−28	−8	−39	−48	−36	94	69	58	4	5	−14
ITC0654-Petite naine-pseudostem	−8	75	85	−46	−55	−70	27	−38	−46	0	5	−14
ITC0101-Fougamou-pseudostem	−23	−11	−13	−14	115	0	102	33	77	2	−15	0
ITC0659-Namwah Khom-pseudostem	−15	68	66	−27	113	−5	30	49	123	89	96	56
ITC0346-Giant Cavendish-corm	55	−19	12	0	100	−7	67	76	0	−19	1	−7
ITC0654-Petite naine-corm	25	−15	48	70	−54	−48	135	13	16	8	−4	6
ITC1138-Saba corm	73	77	−2	−47	−38	−24	43	4	17	98	15	−27
ITC0643-Cachaco-corm	−22	−5	−11	−49	27	48	32	65	31	5	110	−22

E—Ethanol, A—Acetone, H—Hexane. Growth inhibition (%) data were rounded to the nearest integer. Single-underlined values represent inhibition above 50%. All four Gram-negative strains exhibited 100% inhibition when tested with ciprofloxacin (20 µg/mL).

**Table 5:** IC_50_ of selected extracts.

Cultivar, Part, Solvent	Bacteria (IC_50_ Concentration in μg/mL)
ITC1356-Mbwazirume-ethanol	SA (511), BC (130), ML (88), SF (571)
ITC1356-Mbwazirume-acetone	BC (53), ML (33), SF (83), SS (152)
ITC0101-Fougamou-leaf -acetone	BC (315), ML (511), SF (271), SS (99)
ITC0659-Namwah Khom-acetone	SA (1104), BC (190), ML (45), SF (31)
ITC1138-Saba-acetone	SA (373), BC (99), ML (56), SF (37)
ITC0472-Pelipita-acetone	SA (442), BC (116), ML (52), SF (58)
ITC0652-KlueTiparot-ethanol	SA (407)
ITC0652-KlueTiparot-pseudostem-ethanol	AH (1354)
ITC0652-KlueTiparot-acetone	SA (433), BC (190), ML (31), SF (28)
ITC0643-Cachaco-leaf-ethanol	SA (1117), BC (159), ML (287), SF (704), EC (1787)
ITC0767-Dole-leaf-acetone	SA (580), BC (61), ML (144), SF (53), SS (107), AH (1973)
ITC0767-Dole pseudostem-acetone	SA (1239), BC (330), ML (121), SF (319)

Note: IC_50_ values are presented only for selected extracts that gave high inhibition levels. Results of aqueous extracts are not shown as many of these shows microbial growth after further dilution without inoculum, presumably due to endophytes [32]. AH—*Aeromonas hydrophila*, EC—*Escherichia coli*, SS—*Shigella sonnei*, BC-*Bacillus cereus*, ML-*Micrococcus luteus*, SA—*Staphylococcus aureus*, SF—*Streptococcus faecalis*.

**Table 6:** Summary of Candida anti-biofilm activity (BIC_50_ in µg/mL).

ITC Code, Cultivar, Plant Part	Acetone	Ethanol	Hexane	Water
ITC0346-Giant Cavendish-Leaf	51	35	25	27
ITC1356-Mbwazirume-Leaf	60	37	18	11
ITC0654-Petite naine-Leaf	36	56	31	16
ITC0101-Fougamou-Leaf	57	37	73 (220)	22
ITC0659-Namwah Khom-Leaf	55	85 (31)	34	3
ITC1138-Saba -Leaf	51	17	29	45
ITC0472-Pelipita-Leaf	72	22	52	16
ITC0767-Dole-Leaf	90 (71)	92 (51)	74	23
ITC0654-Petite naine-Pseudostem	24	76 (44)	19	32
ITC0101-Fougamou-Pseudostem	66	22	36	23
ITC1138-Saba -Pseudostem	83 (177)	23	25	36
ITC0472-Pelipita-Pseudostem	79	30	25	30
ITC0652-KlueTiparot-Pseudostem	82 (183)	24	31	43
ITC0643-Cachaco-Pseudostem	41	60	31	16
ITC0767-Dole-Pseudostem	76	81	18	20
ITC0101-Fougamou-corm	12	15	61	22
ITC0659-Namwah Khom-corm	59	8	17	24
ITC0643-Cachaco-corm	50	50	24	19

Growth inhibition (%) data were rounded to the nearest integer. Underlined values represent inhibition above 50%. BIC_50_ concentration expressed in µg/mL are shown in round brackets for select extracts.

## Supplementary Materials

The following are available online at https://www.mdpi.com/2304-8158/9/4/435/s1, Figure S1: Regression curve for gallic acid assayed with Folin–Ciocalteu reagent, Figure S2: Spider plot of antimicrobial activity against various pathogens, of extracts from different banana plant parts prepared in three different organic solvents. Figure S3: Two-fold serial dilution of extracts from different banana cultivars against various pathogens, Table S1: Extract yield (mg/mL) when 1 g dried material of each banana cultivar was extracted with 10 mL of four different solvents separately, Table S2: Total phenolic content (gallic acid equivalents, µg/mg) of different extracts.
