# Supplementary material for: Antimicrobial Activity of Selected Banana Cultivars Against Important Human Pathogens, Including Candida Biofilm
Source: Foods. 2020 Apr 4;9(4):435. doi: 10.3390/foods9040435 (PMC7230924; doi:10.3390/foods9040435)
Supplement: Supplementary file 1 [file foods-09-00435-s001.pdf]

Antimicrobial Activity of Selected Banana Cultivars against Important Pathogens, including Candida Biofilm

Ramin Saleh Jouneghani[1‡], Ana Hortência Fonsêca Castro[1,2‡], Sujogya Kumar Panda[1,3*], Rony Swennen[4,5,6], Walter Luyten[1]

Supplementary material

Table S1. Extract yield (mg/mL) when 1 g dried material of each banana cultivar was extracted with 10 mL of four different solvents separately.

ITC code	Name	Leaf				Pseudostem				Corm			
		A	W	E	H	A	W	E	H	A	W	E	H
ITC0767	Dole	2.1	13.8	3.6	3.1	2.9	29.7	3.5	15	2.5	21.7	2.7	17.1
ITC0643	Cachaco	5.8	17.9	5.2	8.8	2.5	27.9	3.1	9	2	17.7	2.2	14.4
ITC1138	Saba	5.3	20.6	3.7	7.7	2.5	32.2	3.9	8.7	1.6	16.8	3.2	8.9
ITC0652	Kluai Tiparot	5.5	21	4	9.3	2.2	25.2	4.3	8.4	1.9	15.7	2.6	9.3
ITC0472	Pelipita	5.8	19.8	4.8	7.9	2.6	29.6	3.8	2.7	1.7	18.4	2.4	14.8
ITC0659	Namwah Khom	4.8	23.3	5.3	15.7	2.5	27	4.1	7.2	2.1	9.6	1.9	4.3
ITC0101	Fougamou	5.7	24.1	6.2	11.4	3.3	27	7.4	4	6.2	16.1	2.5	12.5
ITC0654 *	Petite naine	4.9	17.3	4.3	3.3	3.7	26.8	2.1	7.9	3.7	19.9	2.1	13.3
ITC0346	Giant cavendish	3.7	17.7	4.6	8.2	2.1	22.9	3.4	7.8	1.5	10.1	2	8.1
ITC1356	Mbwazirume	3	12	6	3.6	2	10	4.8	2.7	4.6	8	2.2	1.4

Note: The dried residue of 1 mL extract was re-dissolved in 200 µL of water (for the aqueous extract) or dimethyl sulfoxide (DMSO) for the organic extracts and tested against each pathogen. A- Acetone, W-Water, E-Ethanol, H-Hexane.

Table S2. Total phenolic content (gallic acid equivalents, µg/mg) of different extracts.

		Leaf			Pseudostem		
ITC code	Name	A	E	H	A	E	H
ITC0767	Dole	185	149	146	160	140	ND
ITC0643	Cachaco	246	163	181	109	149	ND
ITC1138	Saba	ND	ND	149	179	ND	ND
ITC0652	Kluai Tiparot	184	ND	ND	152	169	ND
ITC0472	Pelipita	ND	ND	ND	ND	110	72
ITC0659	Namwah Khom	ND	168	ND	ND	ND	ND
ITC0101	Fougamou	147	24.	ND	ND	309	ND
ITC0654*	Petite naine	ND	ND	150	ND	204	ND
ITC0346	Giant cavendish	213	236	ND	76	42	ND
ITC1356	Mbwazirume	178	187	154	135	176	73

Note: The phenolic concentration of extracts was evaluated from a gallic acid calibration curve (shown below) and data are expressed in gallic acid equivalents (GAE) as µg/mg of the crude extract. A-Acetone, E-Ethanol, H-Hexane, ND-Not determined (because extract showed little antimicrobial activity, or insufficient sample was left after bioactivity testing).

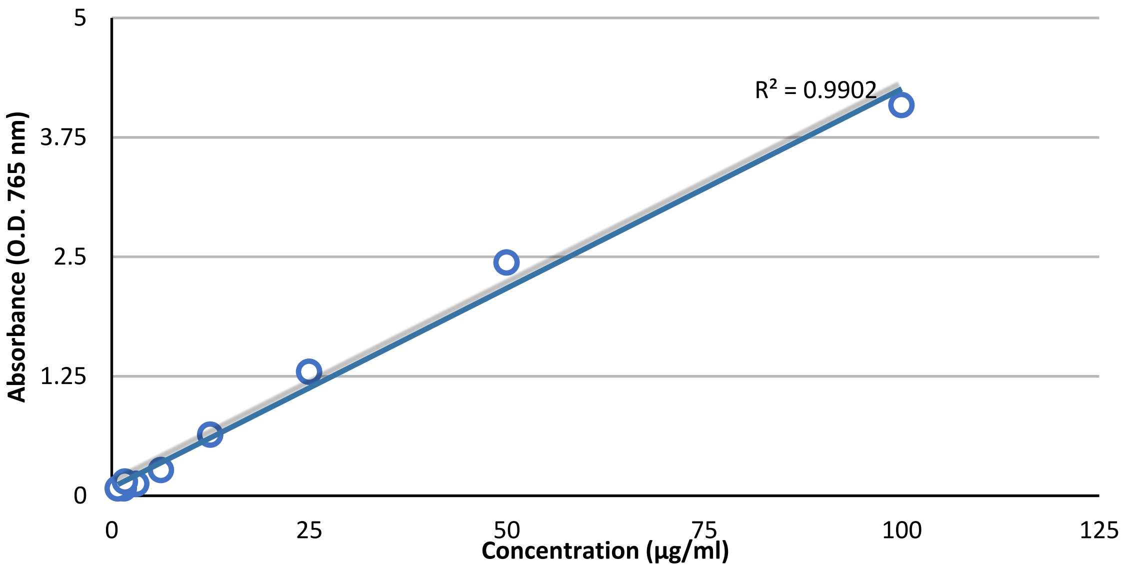

Figure S1. Regression curve for gallic acid assayed with Folin–Ciocalteu reagent. For details, see materials and methods..

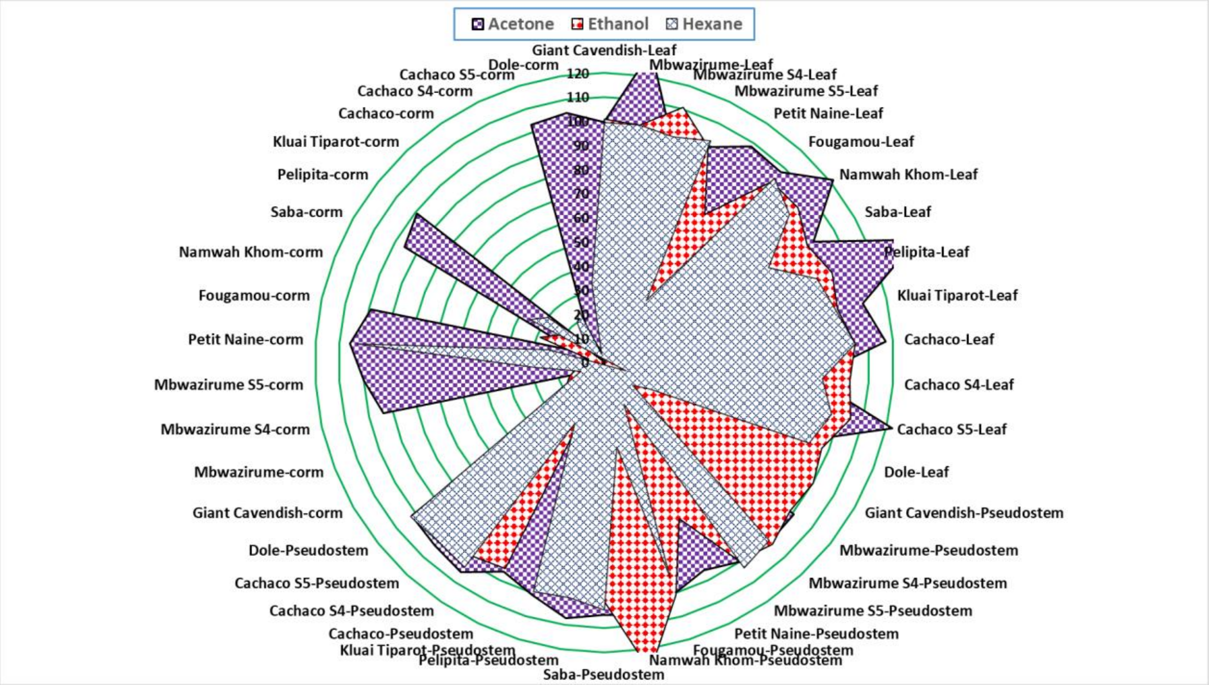

Figure S2. A. Antibacterial activity against *Bacillus cereus.*

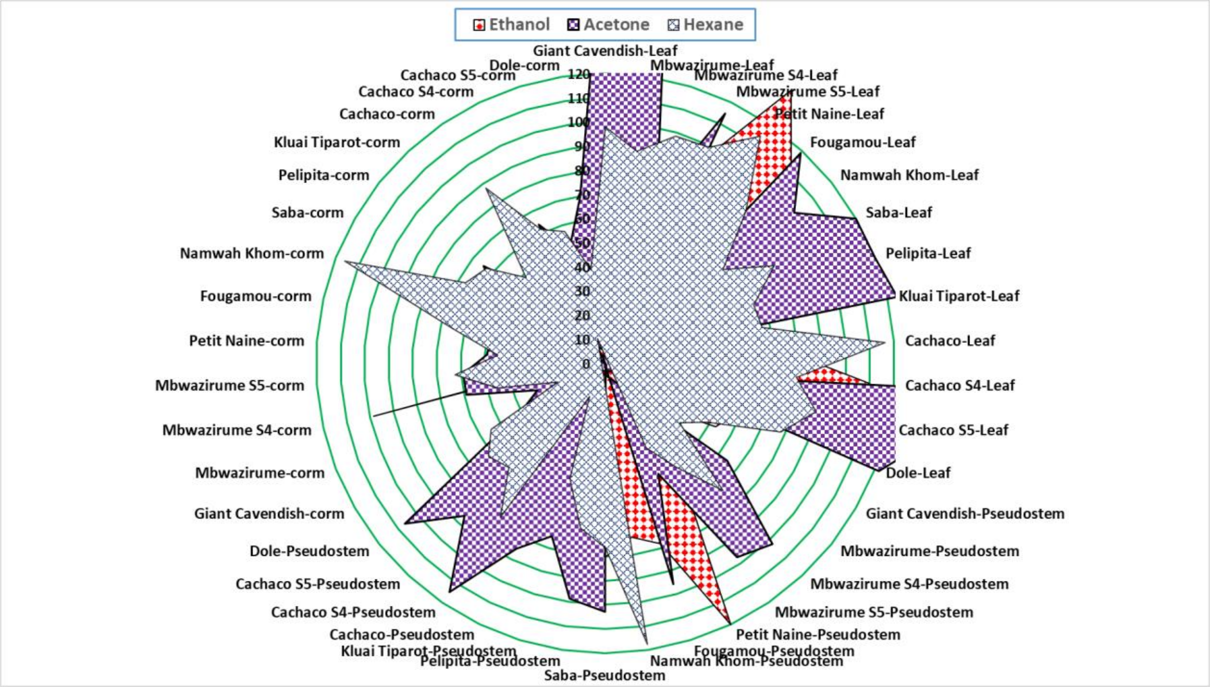

Figure S2. B. Antibacterial activity against *Micrococcus luteus*.

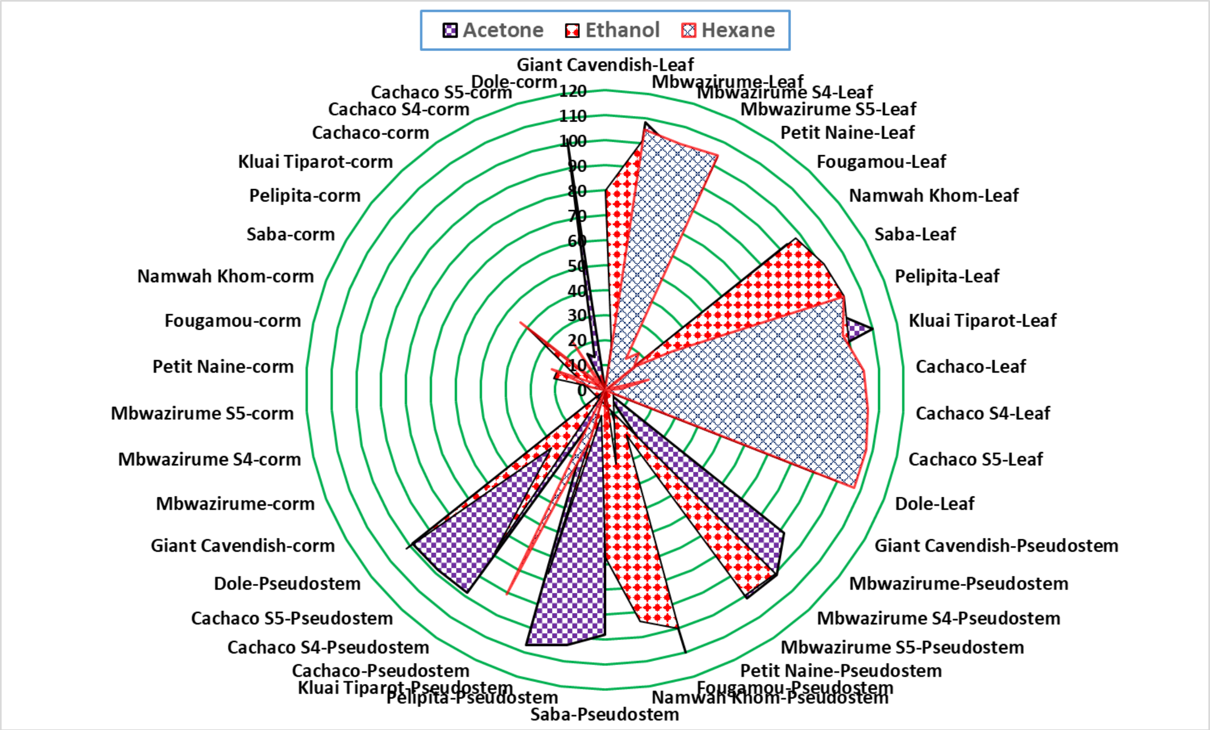

Figure S2. C. Antibacterial activity against *Staphylococcus aureus*.

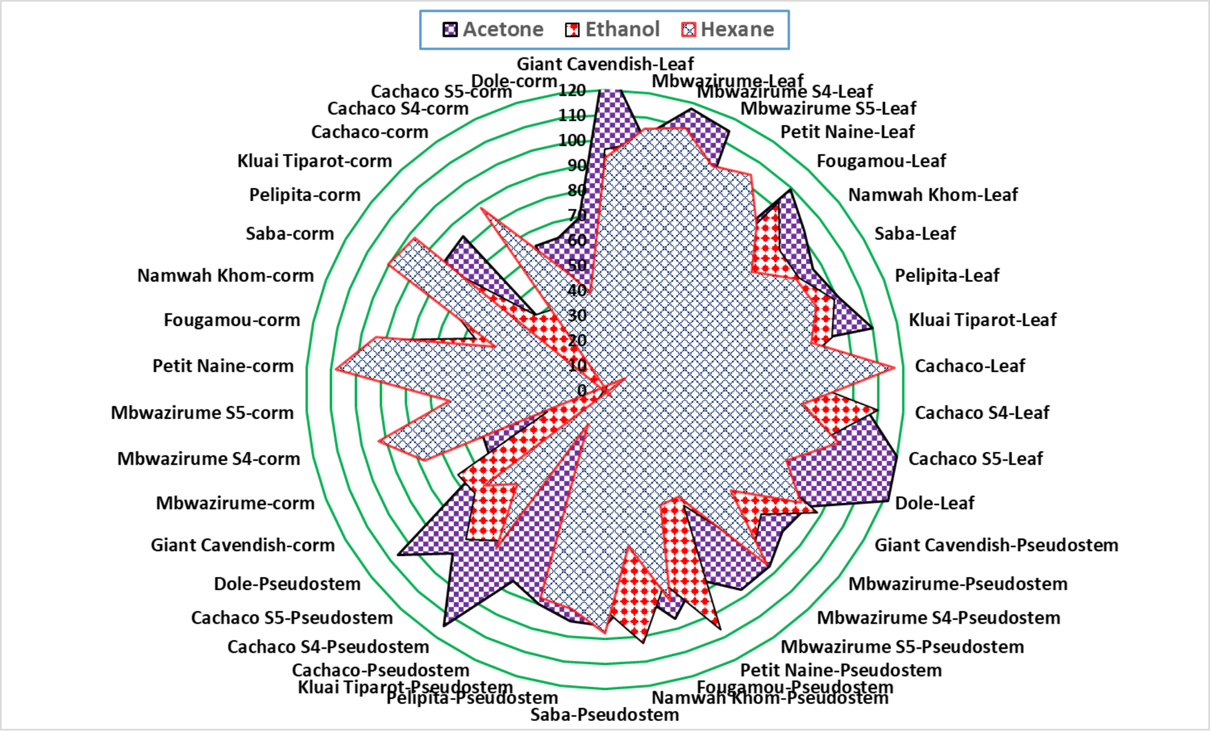

Figure S2. D. Antibacterial activity against *Streptococcus faecalis.*

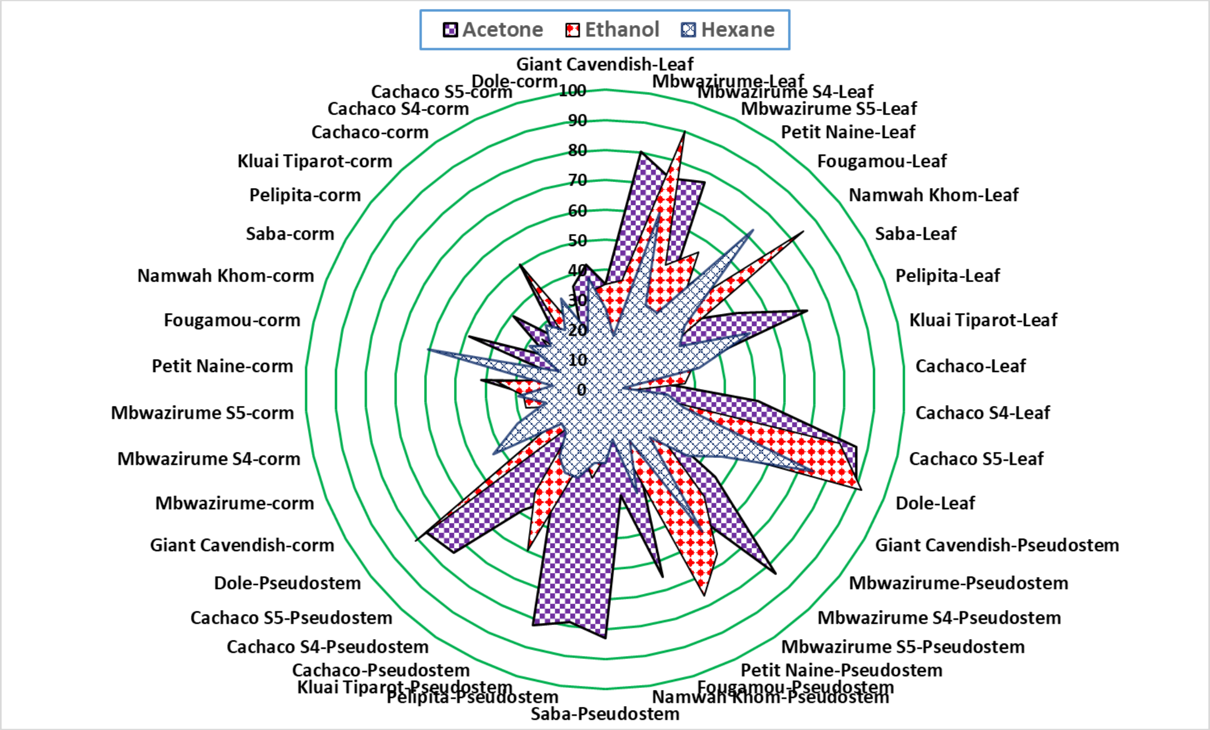

Figure S2. E. Anticandidal activity against *Candida albicans* biofilm.

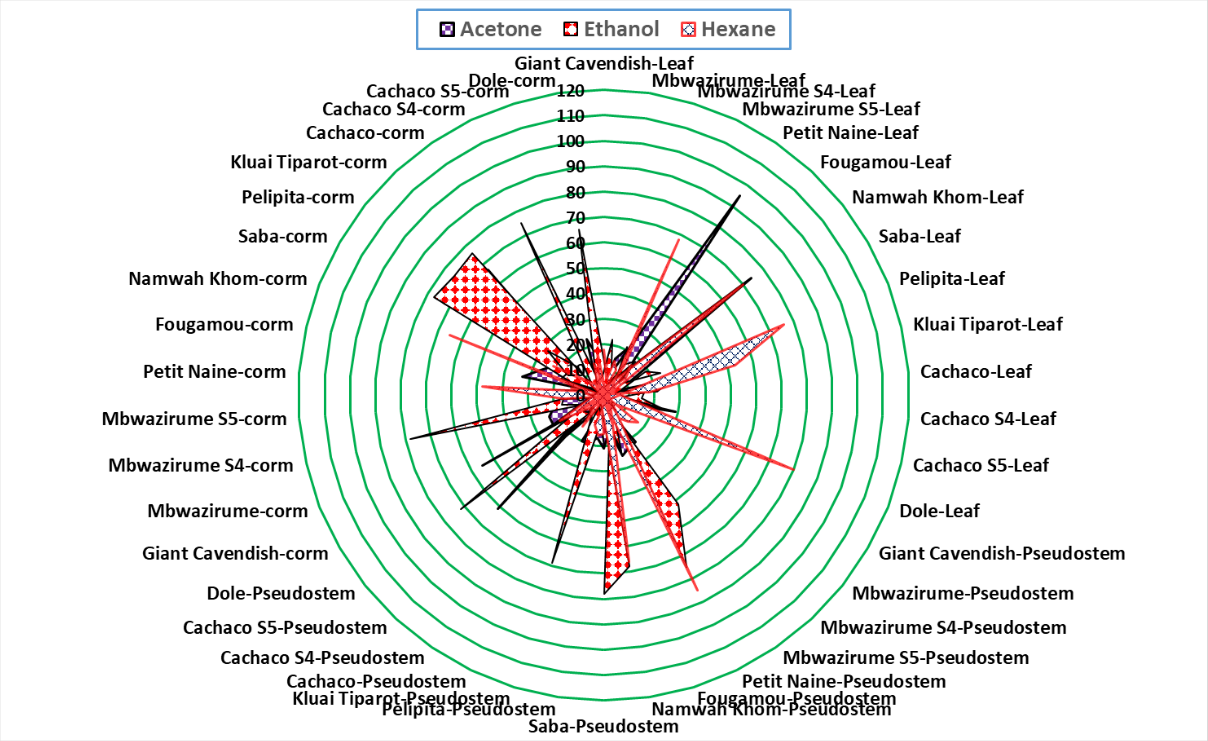

Figure S2. F. Antibacterial activity against *A. hydrophila.*

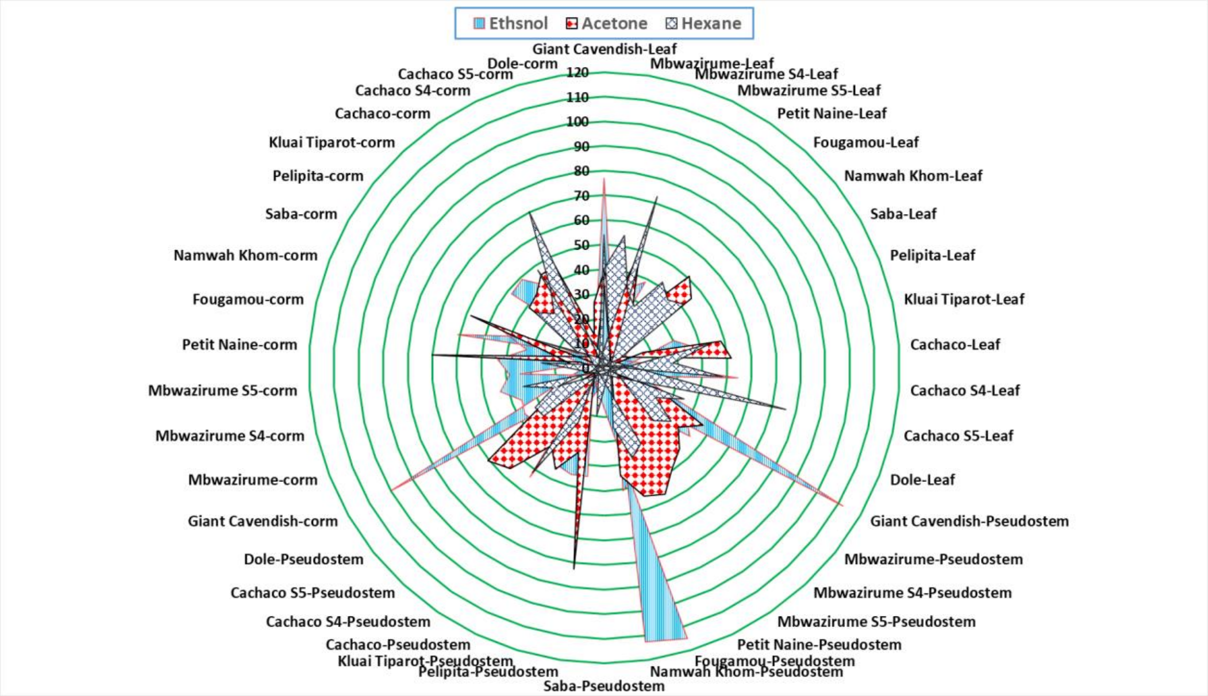

Figure S2. G. Antibacterial activity against *E. coli.*

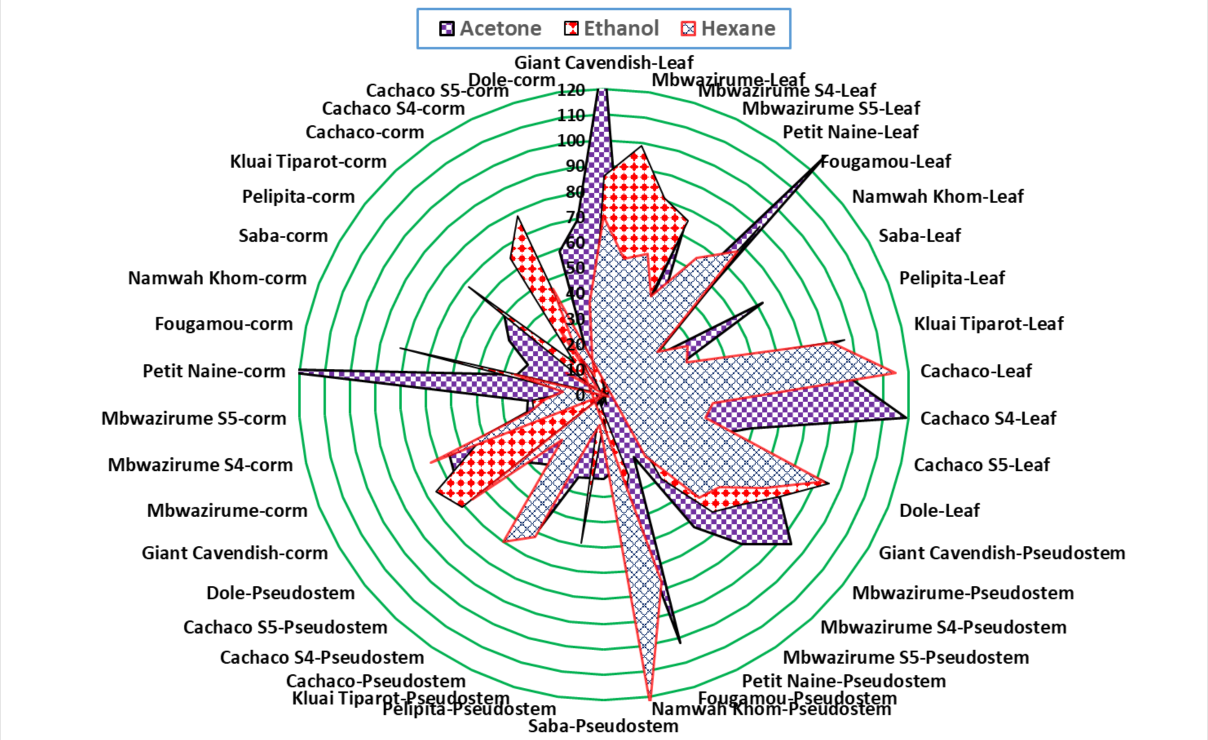

Figure S2. H. Antibacterial activity against *S. enterica.*

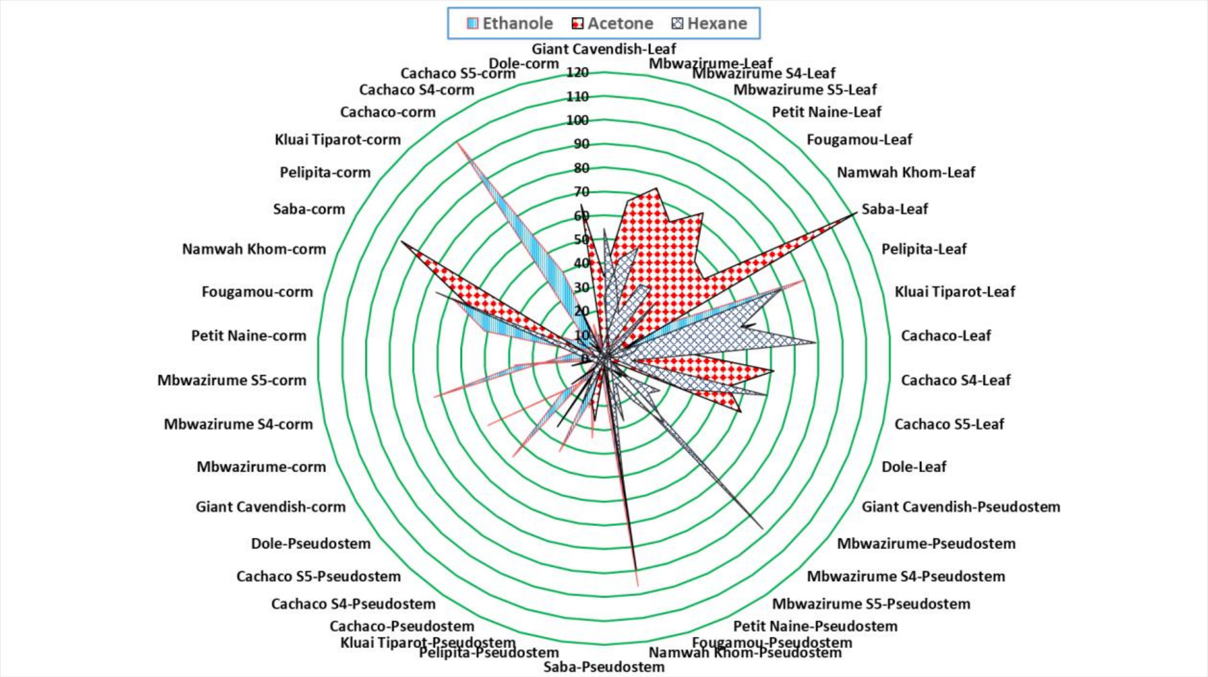

Figure S2. I: Antibacterial activity against *S. sonnei.*

Figure S2. Spider plot of antimicrobial activity against various pathogens, of extracts from differentbanana plant parts prepared in three different organic solvents.

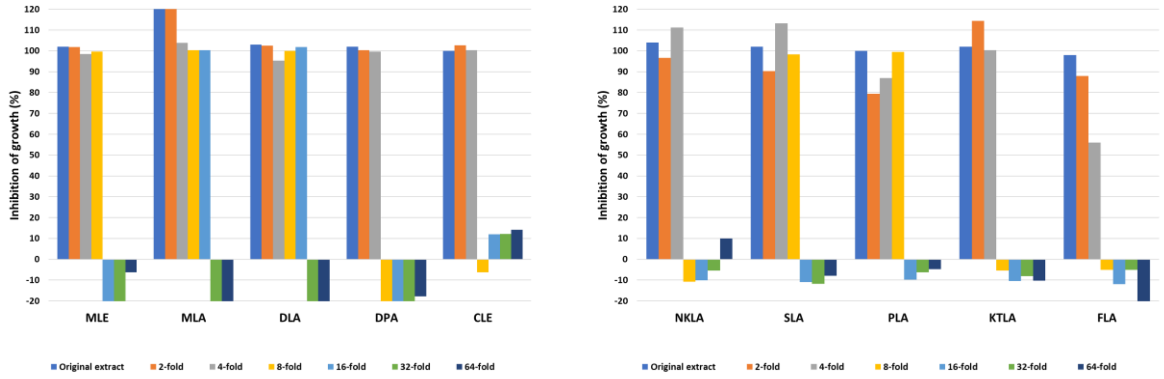

Figure S3. A. Growth inhibition of *Bacillus cereus* by dilution series of selected extracts that show the strongest inhibition (>90% by original extract).

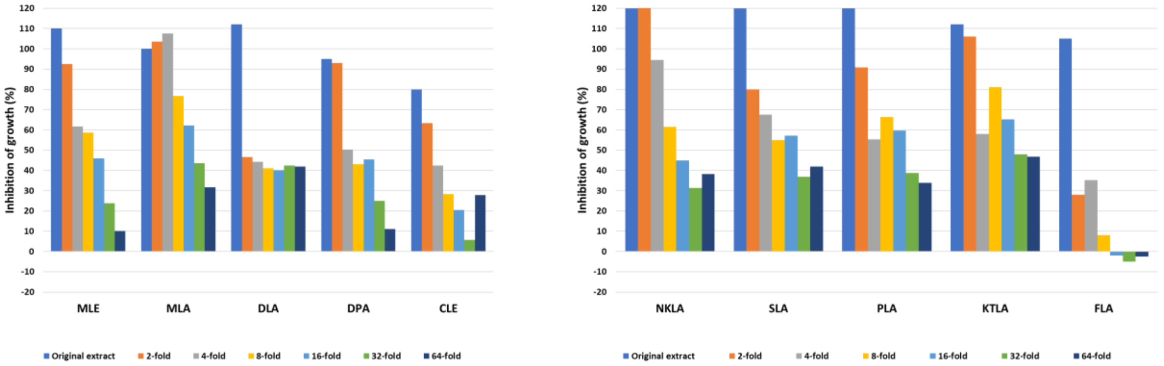

Figure S3. B. Growth inhibition of *Micrococcus luteus* by dilution series of selected extracts that show the strongest inhibition (>90% by original extract).

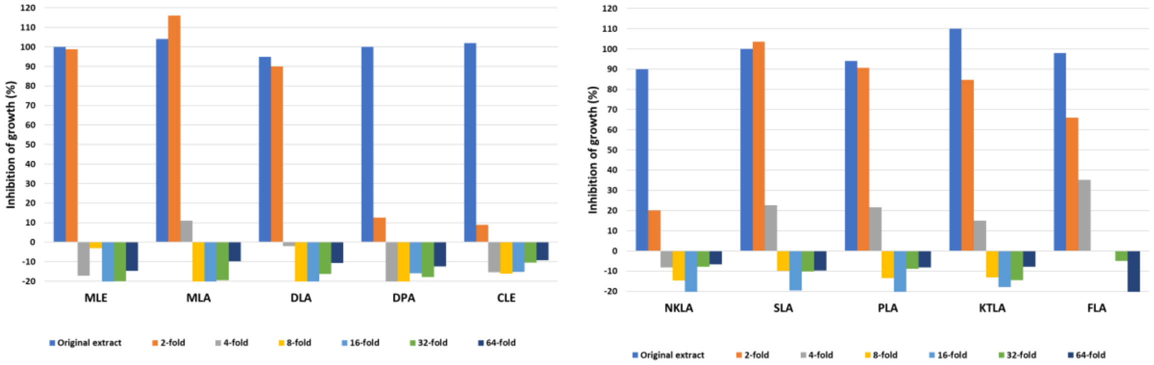

Figure S3. C. Growth inhibition of *Staphylococcus aureus* by dilution series of selected extracts that show the strongest inhibition (>90% by original extract).

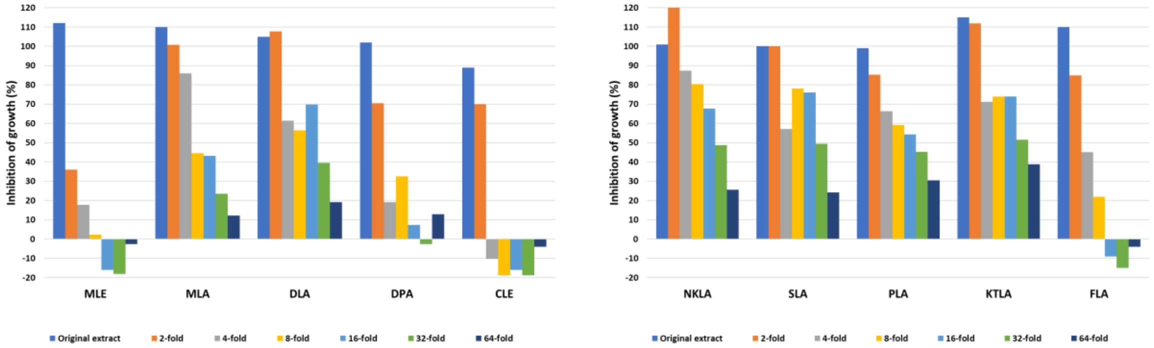

Figure S3. D. Growth inhibition of *Streptococcus faecalis* by dilution series of selected extracts that show the strongest inhibition (>90% by original extract).

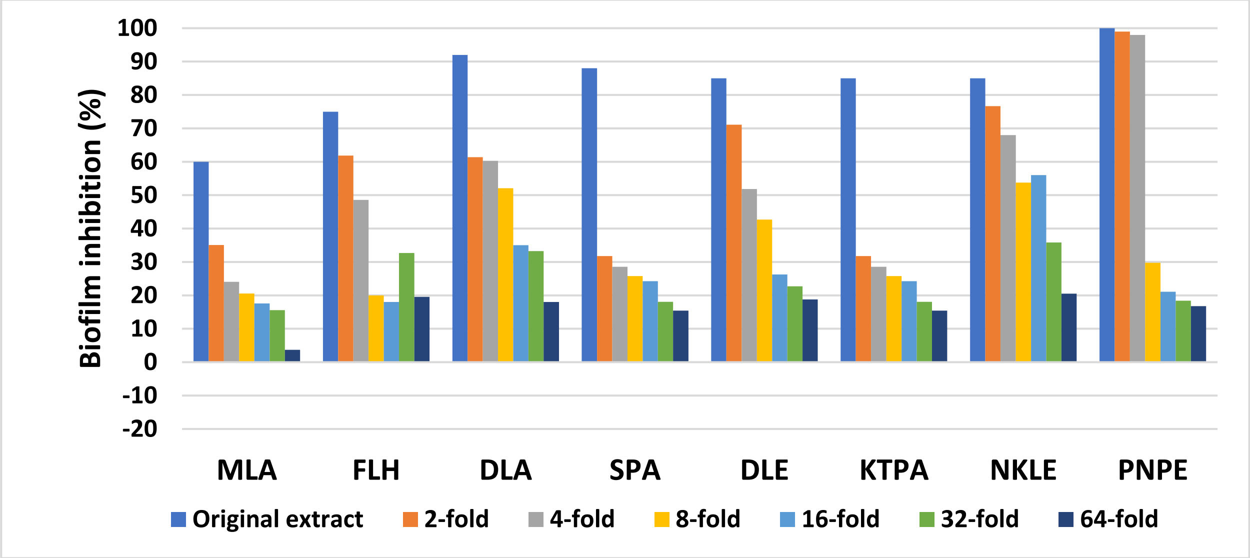

Figure S3. E. Inhibition of *Candida albicans* biofilm formation by dilution series of selected extracts that show the strongest inhibition (>60% by original extract).

Legend: A two-fold serial dilution series was prepared of all original extracts that showed > 90% growth inhibition (>60% biofilm formation in case of *Candida*). Growth inhibition was estimated from OD measurements of the inoculated well treated with the extract (corrected for the contribution of the extract itself), compared to a solvent control (DMSO). For sample codes, see table below.

Code	cultivar, plant part, solvent
CLE	Cachaco, leaf, ethanol
DLA	Dole, leaf, acetone
DLE	Dole, leaf, ethanol
FLA	Fougamou, leaf, acetone
FLH	Fougamou, leaf, hexane
KTLA	Kluai tiparot, leaf, acetone
KTPA	Kluai tiparot, pseudostem, acetone
MLA	Mbwazirume, leaf, acetone
MLE	Mbwazirume, leaf, ethanol
NKLA	Namwa Khom, leaf, acetone
NKLE	Namwa Khom, leaf, ethanol
PLA	Pelipita, leaf, acetone
PNPE	Petite naine, pseudostem, ethanol

SLA	Saba, leaf, acetone
SPA	Saba, pseudostem, acetone
